# When Rhythm Control Backfires: Flecainide-Precipitated Transient Cardiomyopathy in the Setting of Electrical Cardioversion

**DOI:** 10.7759/cureus.108713

**Published:** 2026-05-12

**Authors:** Keyshla Pagan Morales, Dipak Shah, Dustin Feldman

**Affiliations:** 1 Cardiology, Henry Ford Providence Hospital, Southfield, USA

**Keywords:** atrial fibrillation, bradyarrhythmia, cardioversion, conduction suppression, flecainide associated cardiomyopathy, negative inotropy, peri-cardioversion vulnerability, transient cardiomyopathy

## Abstract

Flecainide, a class Ic antiarrhythmic, is a potent sodium channel blocker that slows conduction through the atria, atrioventricular (AV) node, and His-Purkinje system. It is effective for rhythm control in atrial fibrillation (AF) in appropriately selected patients but carries a risk of significant bradyarrhythmia, conduction system suppression, and negative inotropy. Flecainide is generally considered safe in patients without structural heart disease, left ventricular dysfunction, or significant coronary artery disease; however, rare cases of reversible cardiomyopathy have been reported. Current guidelines continue to recommend flecainide for rhythm control in carefully selected patients with AF who do not have structural heart disease, including for cardioversion of recent-onset AF, pretreatment before direct current cardioversion, the “pill-in-the-pocket” approach, and ongoing maintenance of sinus rhythm. This case describes a 73-year-old male with long-standing persistent AF, no structural heart disease, and normal left ventricular systolic function who was started on flecainide following successful AF ablation. Subsequently, he developed recurrent AF and underwent elective cardioversion, which was complicated by profound conduction suppression and likely flecainide-precipitated transient cardiomyopathy in the setting of electrical cardioversion. Prompt recognition and management led to a full recovery, emphasizing the importance of careful patient selection and monitoring when using flecainide, as it may, in rare cases, be associated with or precipitate cardiomyopathy in the peri-cardioversion setting.

## Introduction

Flecainide is a class Ic antiarrhythmic agent with a primary role in rhythm control strategies for atrial fibrillation (AF) in patients without structural heart disease. Its mechanism involves potent inhibition of fast sodium channels, resulting in slowed conduction through the atria, atrioventricular (AV) node, and His-Purkinje system [1]. The American College of Cardiology and American Heart Association, as well as the European Society of Cardiology, recommend flecainide for AF only in patients without significant structural heart disease, prior myocardial infarction, or left ventricular dysfunction, due to increased mortality observed in the Cardiac Arrhythmia Suppression Trial (CAST) [1-3]. In line with current European guidelines, flecainide is recommended for cardioversion of recent-onset AF, pretreatment prior to direct current cardioversion, out-of-hospital acute oral therapy ("pill-in-the-pocket" approach), and ongoing maintenance of sinus rhythm in patients without structural heart disease [1,2,4]. Flecainide is contraindicated in patients with heart failure with reduced ejection fraction (HFrEF), significant ventricular hypertrophy, or ischemic heart disease with active ischemia [1,2,4-6]. While rare, transient cardiomyopathy and severe conduction disturbances have been reported, particularly in the setting of toxicity or concomitant AV nodal blocking agents [1,2,4,5,7-10]. The true incidence of flecainide-associated cardiomyopathy remains unknown. In a retrospective study of over 400 patients treated with class Ic antiarrhythmics, only a handful developed new systolic dysfunction potentially attributable to the drug [11]. Its occurrence specifically in the peri-cardioversion setting, following AF ablation, and in the context of concomitant beta-blocker use represents an even rarer and largely underrecognized clinical scenario, with existing evidence limited to isolated case reports.

## Case presentation

A 73-year-old male with long-standing persistent AF for approximately seven years and two prior unsuccessful cardioversions underwent radiofrequency AF ablation. The procedure was performed using a Tactiflex ablation catheter (Abbott Medical, Plymouth, Minnesota) and Vizigo sheath (Biosense Webster, Irvine, California) with a high-power, short-duration strategy. Pulmonary vein and posterior wall isolation were achieved. Pulmonary vein anatomy demonstrated a left common pulmonary vein with superior and inferior poles (LSPV and LIPV) and separate right superior (RSPV) and right inferior pulmonary veins (RIPV), with electrical activity noted in the RIPV. Ablation lesions were delivered with contact force between 5 and 20 g and radiofrequency power of 50 W for 7 to 10 seconds, with shorter duration applied along the posterior wall and near the esophagus.

An electrophysiology study performed at the conclusion of the ablation showed a sinus cycle length of 1100 ms, PR interval of 186 ms, QRS duration of 82 ms, QT interval of 417 ms, atrium-to-His bundle (AH) interval of 130 ms, and His bundle-to-ventricle (HV) interval of 47 ms. The ventriculoatrial block cycle length was 330 ms with concentric and decremental conduction, and the atrioventricular block cycle length was 310 ms from the coronary sinus.

Flecainide 50 mg twice daily was initiated post-ablation for rhythm maintenance. The patient was continued on metoprolol and rivaroxaban. Several days after the procedure, AF recurred, and the patient subsequently underwent elective electrical cardioversion one month later. The last dose of flecainide was taken the evening prior to the procedure, and metoprolol was taken the morning of cardioversion.

During the cardioversion, a single synchronized 200 J biphasic shock was delivered. Immediately after, the patient developed asystole with associated hypotension. Epinephrine and atropine were administered with supplemental oxygen support. Cardiopulmonary resuscitation was not required, as the patient regained consciousness following medication administration and remained neurologically intact. Subsequently, the patient developed atrial flutter with 2:1 conduction. As the effects of the medications waned, the patient developed a junctional rhythm with bradycardia and recurrent hypotension, requiring additional atropine and epinephrine followed by initiation of a dopamine infusion.

Transthoracic echocardiography demonstrated severe global hypokinesis with a left ventricular ejection fraction of 15% to 20% (Videos 1, 2), despite previously normal systolic function and no known structural heart disease. The patient was admitted to the Cardiovascular Intensive Care Unit (CVICU). Management included discontinuation of flecainide and beta-blockers, intravenous sodium bicarbonate for flecainide-related sodium channel blockade, glucagon for beta-blocker reversal, theophylline for sinoatrial and atrioventricular nodal support, heparin for stroke risk reduction, and electrolyte optimization (potassium >4 mmol/L and magnesium >2 mmol/L). Pacemaker placement was deferred pending clearance of the medications from the patient’s system.

**Video 1:** Apical four-chamber transthoracic echocardiogram demonstrating severely reduced left ventricular systolic function (EF 15–20%) with global hypokinesis and moderate to severe biatrial enlargement, obtained immediately after cardioversion. EF: ejection fraction.

**Video 2:** Apical three-chamber transthoracic echocardiogram demonstrating severely reduced left ventricular systolic function (EF 15–20%) with global hypokinesis, obtained immediately after cardioversion. EF: ejection fraction.

Over the following days, the patient’s hemodynamic status improved, dopamine was successfully weaned, and the rhythm transitioned from junctional rhythm to rate-controlled AF (Figures 1, 2). He was discharged home in stable condition on digoxin 0.125 mg daily and metoprolol tartrate 12.5 mg twice daily. A repeat echocardiogram one month later demonstrated complete recovery of left ventricular function with an ejection fraction of 60% to 65%.

**Figure 1:**
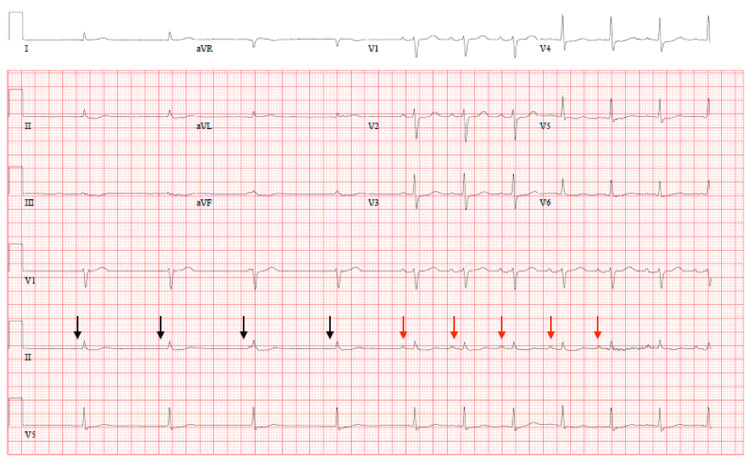
Twelve-lead electrocardiogram obtained in the CVICU demonstrating sinus rhythm (red arrows) with a competing junctional rhythm (black arrows). CVICU: Cardiovascular Intensive Care Unit.

**Figure 2:**
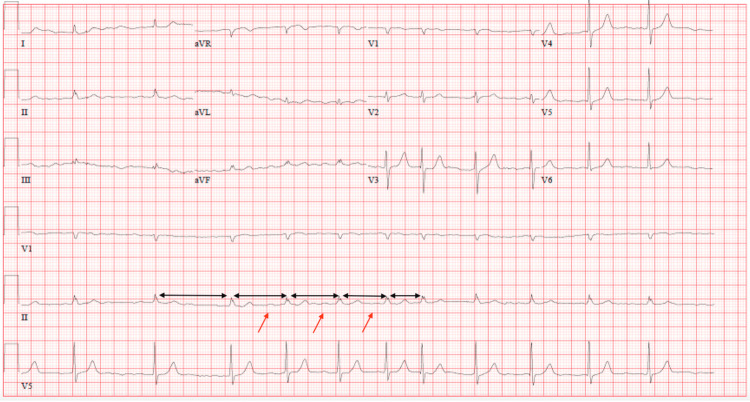
Twelve-lead electrocardiogram obtained prior to discharge demonstrating rate-controlled atrial fibrillation, characterized by an irregularly irregular ventricular rhythm (black arrows) and absence of discernible P waves (red arrows).

## Discussion

This case highlights a rare, serious, and likely underrecognized complication of flecainide therapy: transient cardiomyopathy in the setting of electrical cardioversion, likely precipitated by flecainide and accentuated by profound conduction suppression and negative inotropy, with only a few similar cases reported in the literature.

Flecainide is known to cause sinus arrest, AV block, and asystole, particularly at higher plasma levels or in patients with subclinical conduction disease or concomitant AV nodal blockers [1,2,4-7]. The risk of proarrhythmia and negative inotropy is highest in patients with structural heart disease, ventricular scar, or left ventricular dysfunction, and flecainide is contraindicated in these populations [1,2,5-7,12]. The American College of Cardiology and American Heart Association recommend flecainide only for patients without structural heart disease or significant ventricular dysfunction [1,2,4-6]. The combination of flecainide and beta-blockers can result in additive suppression of the conduction system, increasing the risk of bradyarrhythmia and hemodynamic collapse [1,2,4-7]. The observed reversible cardiomyopathy is consistent with the negative inotropic effects of flecainide, which are dose-dependent [4,7]. Full recovery of left ventricular function after drug discontinuation and supportive care is consistent with prior reports of reversible flecainide-associated cardiomyopathy [1,2,5,7-10].

## Conclusions

Flecainide may precipitate profound bradyarrhythmia and likely contribute to transient cardiomyopathy through conduction suppression and negative inotropy, particularly in the peri-cardioversion setting with concomitant AV nodal blockers or increased peri-procedural vulnerability. The risk is highest in patients with structural heart disease or left ventricular dysfunction, and flecainide should be avoided in these populations. Careful patient selection, dose timing, and peri-procedural monitoring are essential. Clinicians should maintain vigilance for hemodynamic instability and be prepared to provide supportive care, including drug discontinuation and reversal agents. Although rare, flecainide-associated cardiomyopathy is a documented and reversible complication, underscoring the need for awareness and prompt management.
